# A subset of myofibroblastic cancer-associated fibroblasts regulate collagen fiber elongation, which is prognostic in multiple cancers

**DOI:** 10.18632/oncotarget.6740

**Published:** 2015-12-23

**Authors:** Christopher J. Hanley, Fergus Noble, Matthew Ward, Marc Bullock, Cole Drifka, Massimiliano Mellone, Antigoni Manousopoulou, Harvey E. Johnston, Annette Hayden, Steve Thirdborough, Yuming Liu, David M. Smith, Toby Mellows, W. John Kao, Spiros D. Garbis, Alex Mirnezami, Tim J. Underwood, Kevin W. Eliceiri, Gareth J. Thomas

**Affiliations:** ^1^ Cancer Sciences Unit, Faculty of Medicine, University of Southampton, Southampton SO16 6YD, UK; ^2^ Clinical and Experimental Sciences Unit, Faculty of Medicine, University of Southampton, Southampton SO16 6YD, UK; ^3^ Laboratory for Optical and Computational Instrumentation (LOCI), Department of Biomedical Engineering, University of Madison, Wisconsin 53706, USA

**Keywords:** extracellular matrix, collagen, cancer associated fibroblasts, tumor microenvironment, second harmonic generation

## Abstract

Collagen structure has been shown to influence tumor cell invasion, metastasis and clinical outcome in breast cancer. However, it remains unclear how it affects other solid cancers. Here we utilized multi-photon laser scanning microscopy and Second Harmonic Generation to identify alterations to collagen fiber structure within the tumor stroma of head & neck, esophageal and colorectal cancers. Image segmentation algorithms were then applied to quantitatively characterize these morphological changes, showing that elongated collagen fibers significantly correlated with poor clinical outcome (Log Rank *p* < 0.05). We used TGF-β treatment to model fibroblast conversion to smooth muscle actin SMA-positive cancer associated fibroblasts (CAFs) and found that these cells induce the formation of elongated collagen fibers *in vivo*. However, proteomic/transcriptomic analysis of SMA-positive CAFs cultured *ex-vivo* showed significant heterogeneity in the expression of genes with collagen fibril organizing gene ontology. Notably, stratifying patients according to stromal SMA-positivity and collagen fiber elongation was found to provide a highly significant correlation with poor survival in all 3 cancer types (Log Rank *p* ≤ 0.003). In summary, we show that increased collagen fiber length correlates with poor patient survival in multiple tumor types and that only a sub-set of SMA-positive CAFs can mediate the formation of this collagen structure.

## INTRODUCTION

The observation that the extracellular matrix ECM associated with solid tumors is significantly altered compared to normal tissues dates back to Dvorak's seminal description of solid tumors as ‘wounds that do not heal’ [1]. However, it is only in recent years, with technical advances in imaging and 3D cell culture techniques, that we are beginning to appreciate the effect of ECM composition and architecture on tumor cell behavior. For example, the increased tissue tension that develops as a result of desmoplasia around solid tumors causes the activation of signaling pathways involved in mechanotransduction, leading to increased of tumor cells [2]. Elevated interstitial pressure has also been shown to play a role in blunting the efficient delivery of chemotherapeutics to tumors [3].

Fibrillar collagen is the principal structural component of the ECM surrounding solid tumors, and it has been demonstrated that collagen cross-linking by lysyl-oxidase (LOX) enzymes promotes tumor cell invasion, stromal activation and desmoplasia [2, 4, 5]. The morphology of collagen fibers can be analyzed in histological samples using multi-photon laser scanning microscopy (MPLSM) and Second Harmonic Generation (SHG), exploiting the non-centrosymmetric properties of fibrillar collagen to generate highly specific non-linear optical signals and enable the visualization of intrinsic tissue contrast [6, 7]. This has shown that structural changes to collagen fibers, such as alterations in orientation relative to the tumor boundary, can facilitate tumor cell invasion [8, 9]. Furthermore, detection of these changes in tumor specimens have been found to significantly correlate with poor prognosis in human breast cancer patients [6].

Most research into the role of collagen topology in tumor progression has been performed in breast cancer, and few studies have investigated collagen morphology in other tumor types. It is also unclear how structural alterations to fibrillar collagens are regulated. The stromal presence of cancer associated fibroblasts (CAFs) with a myofibroblast phenotype significantly correlates with poor survival rates in multiple tumor types and these cells have been shown to regulate a number of tumor promoting functions [8, 10, 11]. Myofibroblasts are contractile cells that express α-smooth muscle actin (SMA) and secrete ECM proteins, playing a critical role in tissue remodeling and fibrosis/desmoplasia [12, 13]. While many studies have investigated the secretory role of myofibroblasts with regard to cytokines and growth factors (such as IL- 6, HGF and SDF-1) [10, 11, 14, 15], the role of these cells in regulating ECM structure within the tumor microenvironment remains poorly understood.

In this study we use MPLSM and SHG with image segmentation algorithms to quantitatively analyze collagen structure in head & neck squamous cell carcinoma (HNSCC), esophageal adenocarcinoma (EAC) and colorectal adenocarcinoma (CRC). We show that tumors with elongated collagen fibers have significantly poor survival outcome and that formation of this altered collagen structure can be regulated by TGF-β-induced, SMA-positive myofibroblastic CAFs. Notably however, proteomic/transcriptomic analysis of SMA-positive CAFs cultured *ex-vivo* shows significant heterogeneity in the expression of genes associated with collagen fibril organization, and survival analysis reveals tumors containing SMA-positive CAFs that create elongated collagen fibers have a particularly poor prognosis.

## RESULTS

### Collagen fibril organizing gene (CFOG) expression distinguishes between normal and tumor tissues

**Figure 1:**
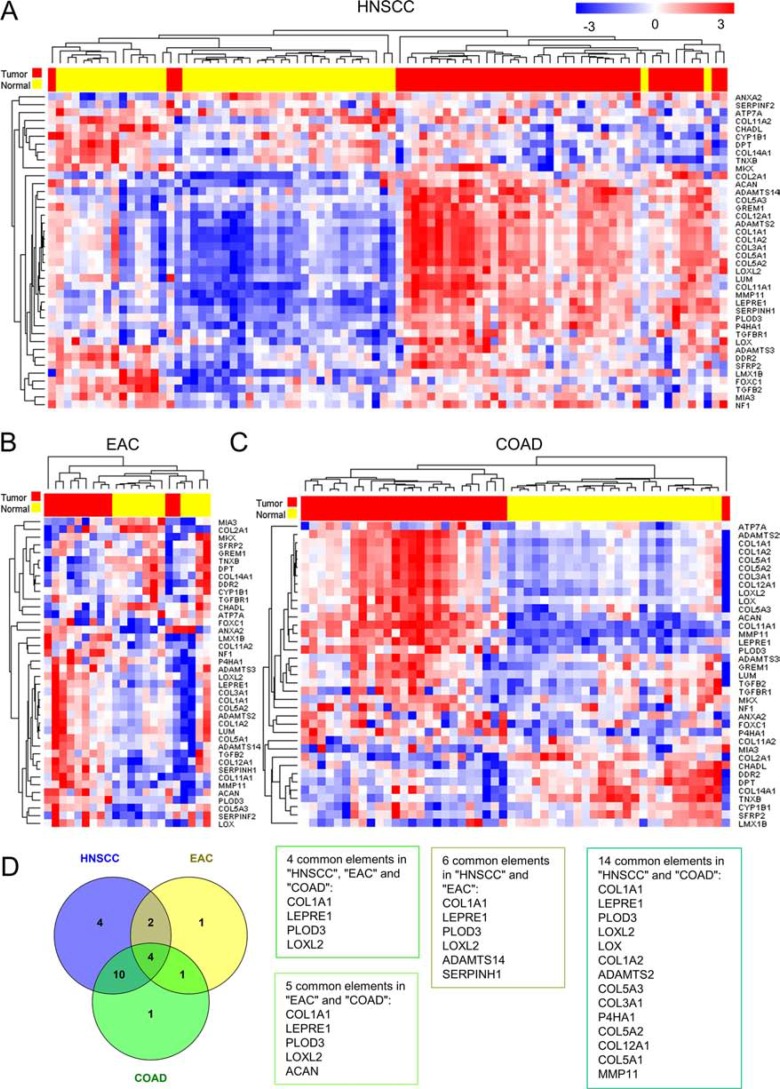
The expression of genes associated with Collagen Fibril Organization Gene (CFOG) ontology (GO:0030199) differentiates between tumor and normal tissue in Head & Neck Squamous cell carcinoma (HNSCC), Esophageal Adenocarcinoma (EAC) and Colon Adenocarcinoma (COAD) RNA Sequencing data from the Cancer Genome Atlas was used to analyze matched tumor and normal samples. (**A–C**) Unsupervised Hierarchical clustering, using a Euclidean distance measure of pairwise average-linkage to determine sample clusters. Expression levels were row normalized for visualization and sample type is shown above the heat map (Yellow = Normal, Red = Tumor). (**D**) Venn Diagram showing the number of these genes significantly up-regulated between tumor and normal tissue (BH adj. *p* < 0.05) and lists summarizing the genes that are commonly up-regulated in different tumor types.

To investigate whether the expression of genes associated with collagen structure was altered in solid tumors, we used publicly available databases for HNSCC, EAC and CRC (TCGA RNASeq). The expression of genes within the collagen fibril organization gene ontology term (GO: 0030199; CFOGs) were analyzed in normal and tumor samples, and unsupervised hierarchical clustering showed that the expression of these genes clearly distinguished between normal and tumor samples in the majority of cases (Figure 1A–1C).

Notably, comparative marker selection analysis identified a number of common genes significantly up-regulated in all cancer types (BH adj. *p* < 0.05; Figure 1D). The proteins encoded by these genes play a critical role in each step of the production and maturation of fibrillar collagens: enzymes regulating lysine and proline hydroxylation (PLOD3, LEPRE1); cross-linking of collagen fibers (LOXL2); and the predominant fibrillar collagen found in tumor stroma (COL1A1).

These data suggest that alterations in the production and organization of fibrillar collagens is an important event in the progression of HNSCC, EAC and CRC.

### SHG imaging reveals structural changes to stromal collagen in tumor tissues

**Figure 2:**
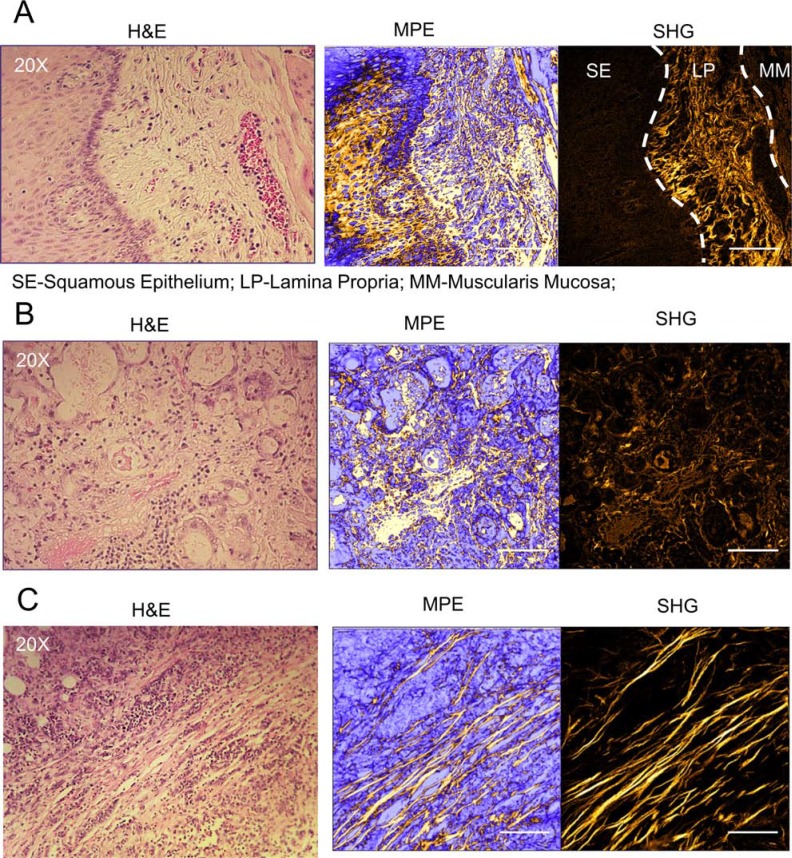
Aligned and elongated collagen fibers are found in the tumor microenvironment Representative images of collagen fiber morphology in normal squamous epithelium/sub-epithelial stroma (**A**) and sub-mucosal tissue (**B**) from esophageal tissue samples. (**C**) Representative image of aligned and elongated collagen fibers in esophageal adenocarcinoma (EAC) tissue, micrographs are shown from hematoxylin & eosin (H & E) stained sections imaged using multi-photon laser scanning microscopy (MPLSM): unfiltered 890 nm multi-photon excitation (MPE); and 445 nm filtered image collecting second harmonic generation (SHG) signal only (fibrillar collagen). Scale Bars represent 100 μm. Corresponding low magnification images are shown in Figure S1.

Since the expression of a number of CFOGs were found to be up-regulated in the HNSCC, EAC and CRC tissues, we used SHG to image alterations in fibrillar collagen morphology in normal and malignant human tissues. In normal tissue, varying degrees of SHG signal were detected in different regions of the mucosa and submucosa (Figure 2A–2B). In areas of squamous epithelium and muscle, relatively low levels of SHG signal was detected; whereas in sub-epithelial stromal regions a strong SHG signal was detected, identifying abundant fibrillar collagen, which consisted of short, ‘curly’ and randomly orientated fibers. Notably, in a subset of tumor cases, a clearly altered collagen stromal structure was observed where collagen fibers were elongated and organized in parallel (Figure 2C). However, not all tumors contained this alteration in collagen morphology, nor was it observed in normal tissues, despite the abundance of fibrillar collagen.

### Quantification of collagen fiber length and alignment

**Figure 3:**
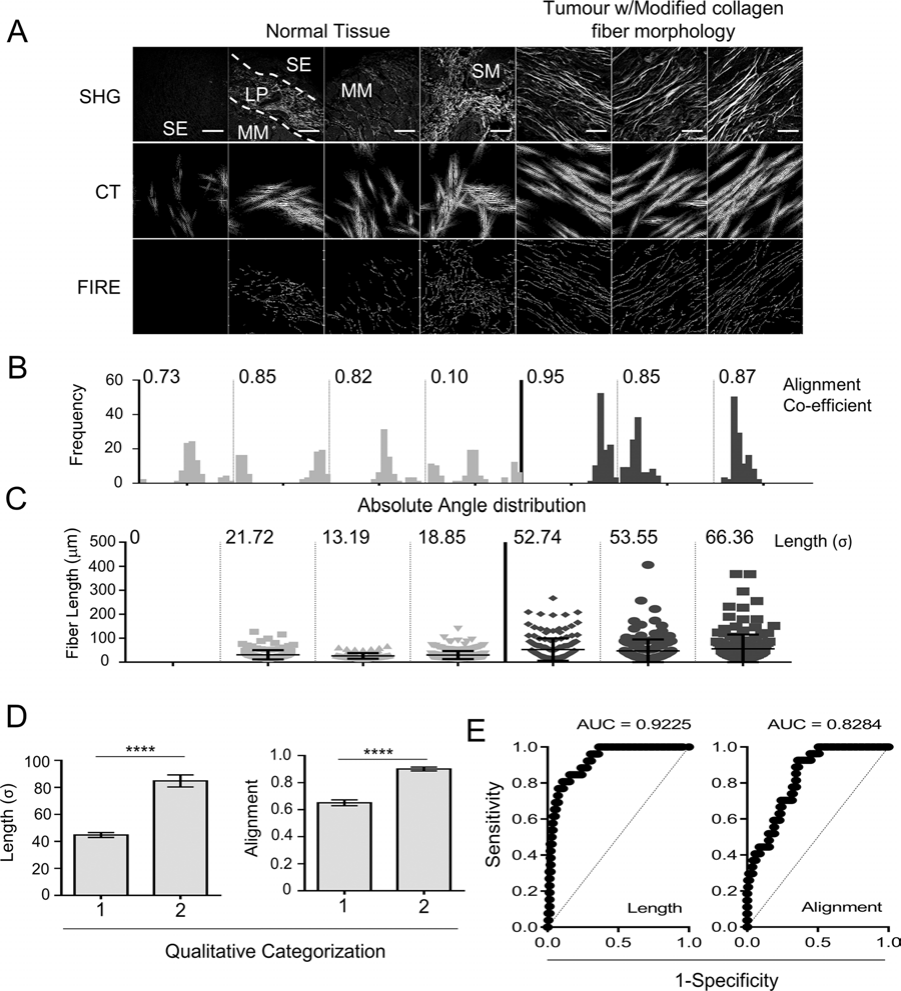
Automated segmentation and quantification of collagen fiber length and alignment can identify the modified collagen stroma observed in tumor tissue (**A**) Image segmentation, SHG images from normal esophageal tissue and EAC are used to identify collagen fibers using curvelet transformation (CT) and fiber extraction (FIRE) algorithms. Reconstructed images are shown for each SHG image. Scale bars represent 100 μm. (**B**) Histograms showing the distribution of absolute fiber angle in each SHG image, measured following CT image segmentation, the alignment co-efficient is also shown for each image. (**C**) Dot plot showing the length of each individual fiber within each SHG image measured using FIRE segmentation; error bars represent the mean ± SD. The standard deviation (σ) of fiber length is shown above each panel. (**D–E**) SHG images of stromal regions from 162 cores of an esophageal cancer tissue microarray were qualitatively assessed for modified collagen morphology (1 = cores without evidence of aligned and elongated collagen fibers; 2 = cores with evidence of aligned and elongated collagen fibers). Histograms show the mean ± SEM for measures of fiber length (σ) (D) and alignment co-efficient (E), statistical significance between groups is assessed by 2-tailed homoscedastic *t*-test ****p < 0.0001. (E) ROC curves showing the sensitivity and specificity for each metric to classify SHG images based on qualitative assessment.

We then quantitatively analyzed collagen structure based on SHG images as described previously [16]. Accurate segmentation of collagen fibers was achieved using a curvelet-denoising filter (CT) [17] followed by an automated tracking algorithm (CT-FIRE) [16] to isolate individual fibers (Figure 3A).

CT segmentation of SHG images was used to analyze the orientation of collagen fibers within a given image by calculating the vector sum of absolute fiber angles, termed the alignment coefficient (Figure 3B). CT-FIRE segmentation was used to measure individual fiber lengths: this showed that in all cases the majority of collagen fibers were relatively small; however a number of elongated fibers were found in tumor samples (Figure 3C). This morphological change was therefore most appropriately described by analysis of the variance in fiber length; and the standard deviation (σ) was used as a rudimentary measure of this change in collagen morphology.

In order to validate the effectiveness of these quantitative metrics at identifying regions where collagen fibers were visibly altered, we compared each quantitative metric with subjective (qualitative) assessment of collagen morphology in images from a single tissue microarray (TMA; 162 cores). Each quantitative measure was significantly up-regulated (*p* < *0.0001*) in cases subjectively identified as having a modified stroma (with aligned and elongated collagen fibers) (Figure 3D); and the quantitative metrics were found to be significant classifiers of this phenotype using receiver-operating characteristic (ROC) analysis (*p* < *0.0001*) (Figure 3E). This shows that the objective quantitative measures, accurately identify the modified collagen phenotype observed by eye.

### Increased collagen fiber length significantly correlates with poor survival rates

**Figure 4:**
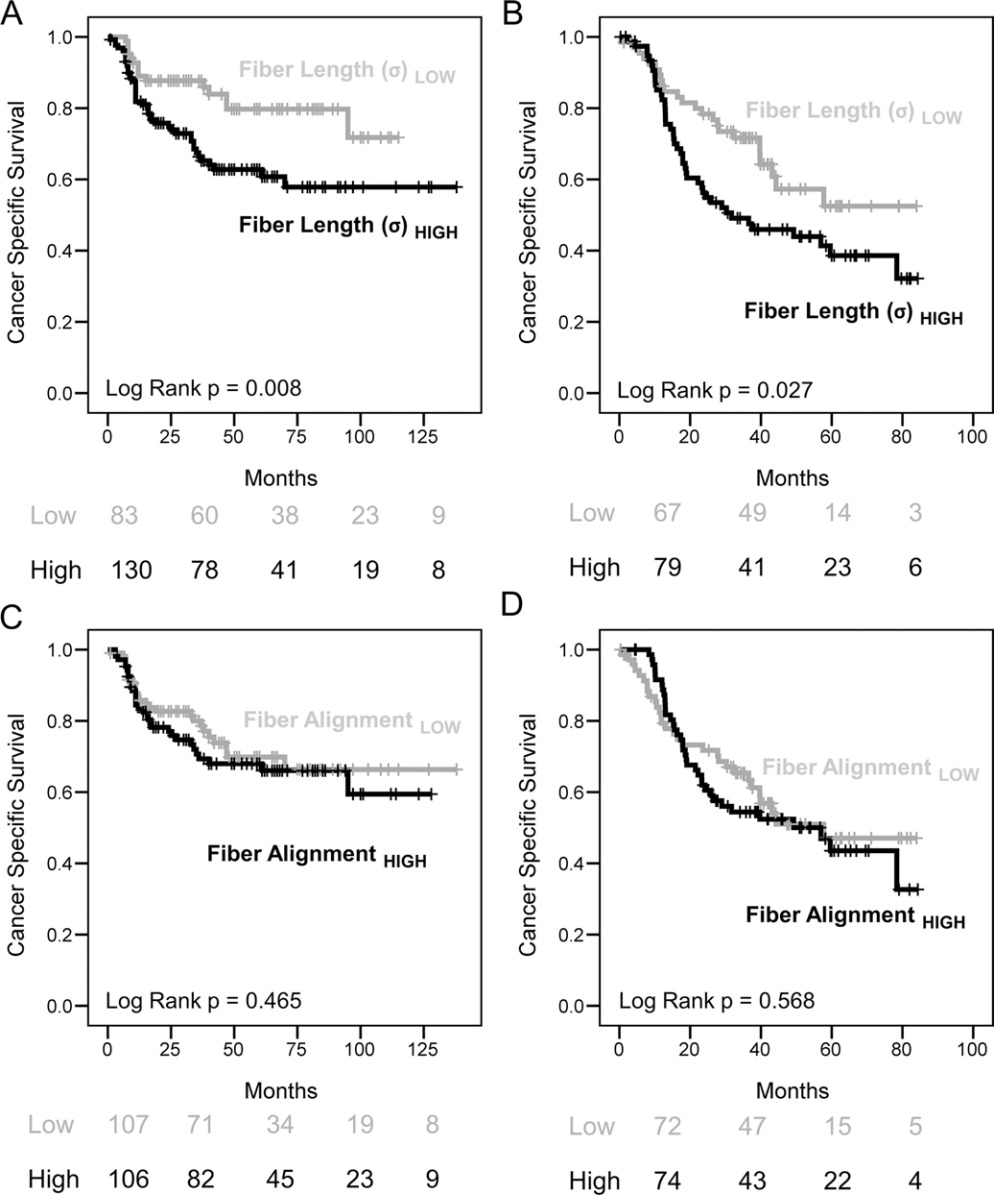
Increased collagen fiber length but not alignment correlates with poor survival rates in HNSCC and EAC (**A–D**) Kaplan Meier (KM) plots showing cancer specific survival rates with tables showing the number of patients at risk at 0, 2, 4 and 6 years are shown below. Quantitative measures of collagen fiber morphology were used to stratify patients showing that increased collagen fiber length correlates with significantly poorer survival rates (Log-rank *p* < 0.05). Multivariate survival analysis is shown in Figure S2.

We next examined the relationship between collagen structure and prognosis using TMAs generated from cohorts of HNSCC and EAC patients (*n* = 213 and 146 respectively [18, 19]; Supplementary Table 1). Representative stromal regions were imaged from multiple tumor cores and the mean value for each patient was used for survival analysis. This showed that patients with increased collagen fiber length had significantly poorer rates of cancer-specific survival and a two-fold increased risk of dying from disease (Figure 4A–4B; HNSCC Log-rank *p = 0.008*, HR = 2.172 [95% CIs: 1.208 to 3.907]; and EAC Log Rank *p* = 0.027, HR = 1.748 [95% CIs: 1.059 to 2.885]); whereas increased fiber alignment did not significantly predict for patient survival (Figure 4C–4D). Multivariate analysis (correcting for tumor stage and grade) showed that fiber length was an independent prognostic factor in HNSCC (HNSCC: HR = 2.137, 95% CI [1.15–3.97], adj. *p* = 0.016) but not EAC (HR = 1.178, 95% CI [0.68–2.06], adj. *p* = 0.564) (Figure S2).

**Figure 5:**
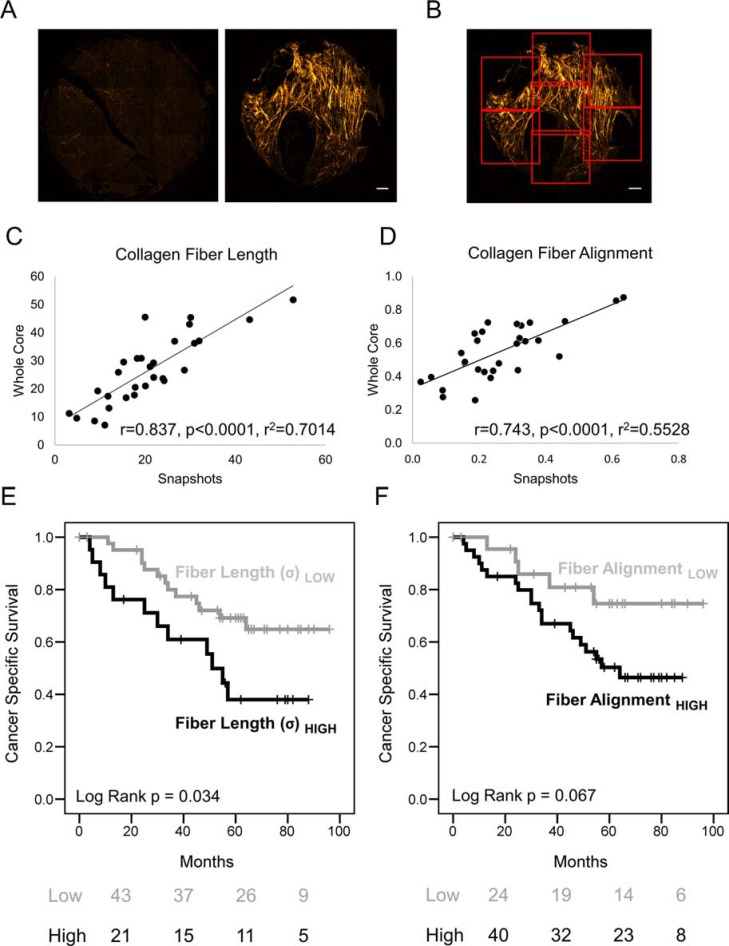
Fully automated measurement of collagen fiber morphology shows increased length correlates with poor prognosis (**A**) Example of stitched SHG images from an entire tumor core with and without evidence of collagen fiber deposition and elongation. Scale Bar represents 100 μm. (**B**) Depiction of automated segmentation of cores into ROIs for analysis and quantification. (**C–D**) Linear regression analysis comparing TMA core quantification using single ‘snapshot’ images of stromal regions or automated segmentation (as described in (B)): a CRC TMA (*n* = 29 patients) was analyzed using both methods and compared. (**E-F**) Kaplan Meier (KM) plots showing cancer specific survival rates with tables showing the number of patients at risk at 0, 2, 4 and 6 years. Quantitative measures (using the automated segmentation method) of collagen fiber morphology were used to stratify patients, showing that patients with increased collagen fiber length have significantly poorer survival rates (Log-rank *p* < 0.05). Multivariate survival analysis is shown in Figure S2.

To investigate the correlation of collagen fiber length with patient survival further, a fully automated procedure was employed to analyze a CRC patient cohort with stage I/II disease (*n* = 64), where patients had received uniform treatment and either developed metastases within 5 years (*n* = 32) or remained metastasis free (*n* = 32; described previously in Bullock et. al [20]). Automated slide scanning and image stitching was employed to image the entire TMA, each tumor core was then split into 7 regions of interest ROIs (Figure 5A–5B) and each ROI was analyzed using the image segmentation algorithms described above. The maximum value for collagen fiber length or alignment within each core was determined and, as above, the mean value for each patient was used for survival analysis. This automated method was compared to the ‘snapshot’ imaging approach employed with the HNSCC and EAC TMAs and a highly significant correlation was observed (*r* = 0.837, *p* < 0.0001, *r*^2^ = 0.7014; Figure 5C–5D). As with the other tumor types this showed that patients with increased collagen fiber length had significantly poorer rates of cancer-specific survival with a two-fold increased risk of dying (Figure 5E; Log-rank *p* = 0.034, HR = 2.282 (95% CIs [1.040 to 5.007])). This analysis also showed that increased fiber alignment indicated poor patient survival to a level approaching significance (Figure 5F; Log-rank *p* = 0.067). Similar to HNSCC, multivariate analysis showed that fiber length was an independent prognostic variable (stage and grade adjusted) in CRC (HR = 3.226, 95% CI [1.27–8.18], adj. *p* = 0.014).

### Myofibroblasts/CAFs regulate collagen morphology within the tumor microenvironment

**Figure 6:**
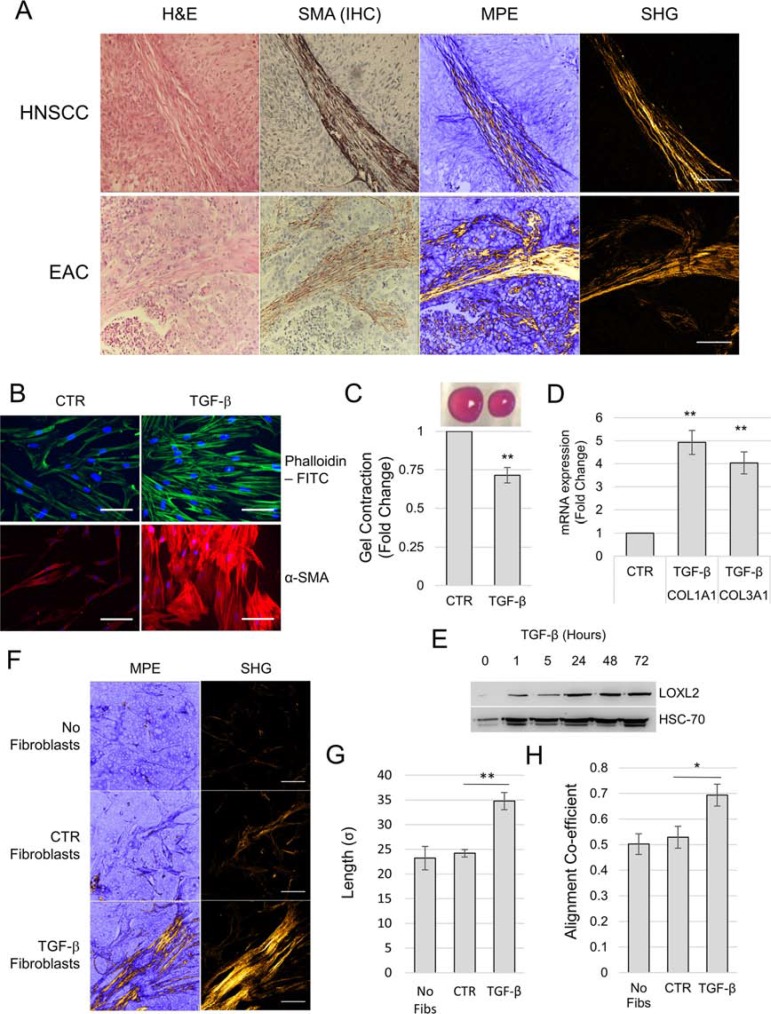
Myofibroblasts/CAFs generate aligned and elongated collagen fibers *in vivo* (**A**) HNSCC and EAC tissue stained for α-SMA to denote myofibroblasts/CAFs and serial H & E stained sections imaged for SHG. Scale bar represents 100 μm. (**B–D**) Treating human fetal foreskin fibroblasts (HFFF2) with TGF-β (2 ng/ml for 72 hours) induces fibroblast-to-myofibroblast differentiation. (**B**) Micrographs showing phalloidin staining of filamentous actin and immunocytochemistry staining for α-SMA. (C) Collagen gel contraction assay: above, representative image of collagen gels with control or TGF-β treated fibroblasts embedded; below, histogram showing gel area measurements from 3 independent experiments, data is presented as mean ± SEM. (D) qPCR measurement of mRNA expression in TGF-β treated fibroblasts, presented as mean ± SEM from 3 independent experiments. (**E–F**) Analysis of 5PT xenograft tumors in RAG1^−/−^ mice co-injected with fibroblasts treated as indicated. (E) Micrographs showing representative MPLSM and SHG imaging of each condition. (**F**) Quantification of collagen fiber morphology in xenograft tumors. Data is shown as the mean ± SEM from 5 tumors per condition. In all cases statistical significance was assessed by 2-tailed homoscedastic *t*-test (*p < 0.05, **p < 0.01, ***p < 0.001). Scale bars represent 100 μm. Low Magnification images to supplement panel A and F are shown in Figures S3 and S4 respectively.

It has been shown previously that CAFs with a myofibroblastic (SMA-positive) phenotype promote tumor progression and that their presence correlates with poor survival rates in multiple tumor types [18, 21, 22]. A key function of myofibroblasts in normal physiology is to secrete and manipulate ECM proteins as part of the wound healing process [12]. Therefore, we investigated the role of myofibroblasts/CAFs in regulating intratumoral collagen morphology. Immunohistochemical IHC staining of tumors for the myofibroblast marker SMA showed clear co-localization of positive cells with elongated collagen fibers (Figure 6A), suggesting that these cells may regulate collagen fiber elongation. To examine this, we used an *in vivo* HNSCC xenograft (5PT cell line) model where tumor cells were implanted with myofibroblasts or control fibroblasts. Myofibroblasts were generated by treating fibroblasts (HFFF2) *in vitro* with human recombinant TGF-β1 for 72 hours, and transdifferentiation confirmed by increased SMA/cytoplasmic actin stress fiber formation; cellular contractility; up-regulation of fibrillar collagens and LOXL2 expression (Figure 6B–6E).

5PT cells were either injected alone or in combination with control fibroblasts or myofibroblasts and after 5-weeks the tumors were collected. The persistence of HFFF2 over time in this xenograft model was confirmed using PCR for human vimentin (hVIM); hVIM was not detected in murine fibroblasts (C2TF) or xenograft tumors where HFFF2s had not been co-injected with tumor cells (Figure S4A). Additionally, the development of a myofibroblastic stroma in tumors containing TGF-β treated HFFF2s was confirmed by IHC staining for SMA (Figure S4B–S4C). Collagen morphology within these tumors was then analyzed using SHG imaging. This showed that in tumors implanted with myofibroblasts, there were significant increases in collagen fiber length and alignment compared with tumors co-injected with control or no fibroblasts (Figure 6F–6H).

### The SMA-positive CAF population is heterogenic for the expression of proteins associated with collagen fibril organization

**Figure 7:**
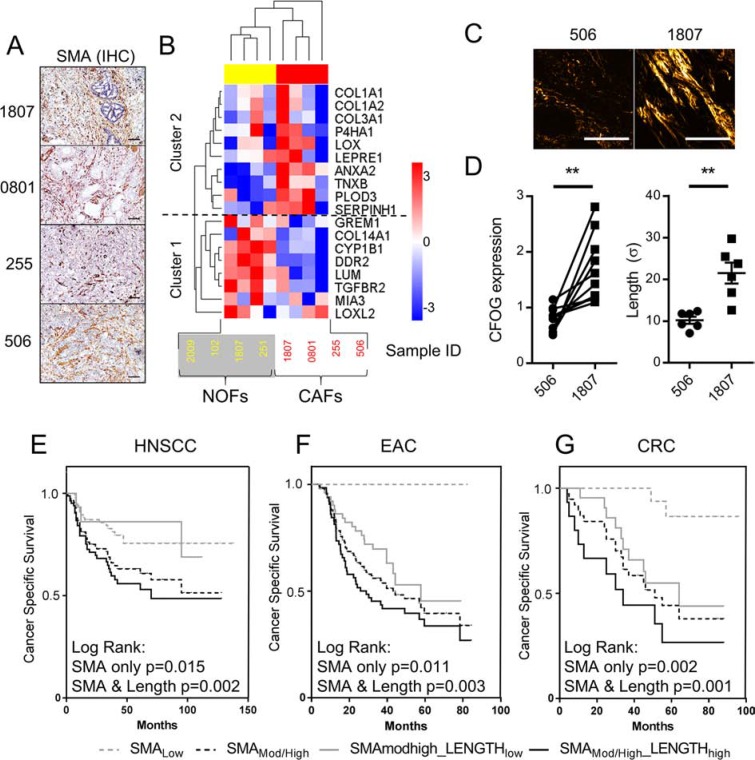
CAFs exhibit significant heterogeneity in the ability to manipulate collagen structure, critically determining patient survival rates (**A**) SMA IHC images showing the tumors from which CAFs were extracted for further analysis. Scale bars represent 100 μm. (**B**) LC-MS analysis of adult esophageal normal and CAFs (NOF (yellow) and CAF (red) respectively). Unsupervised hierarchical clustering, using a Euclidean distance measure of pairwise average-linkage, based on protein expression of genes with collagen fibril organization gene ontology (CFOGs). Expression levels are row normalized for visualization. All proteins were identified with 99% confidence. (**C**) Representative images of SHG imaging in the tumors from which CAF 1807 and 506 were extracted. (**D**) Graphs showing the difference in relative expression of each CFOG within cluster 2 and collagen fiber length (mean ± SEM from 6 stromal ROIs in each tumor) between CAF 506 and 1807, statistical significance was assessed by 2-tailed homoscedastic *t*-test **p < 0.01.(**E–G**) Kaplan Meier (KM) plots showing cancer specific survival rates in patients stratified dependent on stromal α-SMA expression (Myofibroblasts/CAFs), measured by immunohistochemistry (IHC) and collagen fiber elongation. See also Figure S5.

Our previous studies using these patient cohorts showed that moderate/high stromal SMA expression predicted for poor survival in each tumor type [10, 18], and analysis of the TMAs constructed for SHG analysis produced similar results (Figure 7E–7G). Our observations that elongated fibers co-localized with SMA-positive cells in human tumors, suggested that collagen fiber length could be used as a surrogate marker for an SMA-positive stroma. However, further analysis showed that intriguingly, increased collagen fiber length was observed in only a sub-set of SMA-positive cases (75%, 57% and 40% in HNSCC, OAC and CRC respectively; Supplementary Table 2). These percentages did not significantly alter when the degree of SMA positivity was subdivided into moderate and high groups, indicating that the development of elongated fibers is not simply a reflection of myofibroblast numbers. This suggests a degree of heterogeneity within the SMA- positive myofibroblast population, where the ability to generate elongated collagen fibers is limited to a sub-set of cells.

To examine collagen-forming heterogeneity within the myofibroblastic CAF population we used multidimensional liquid chromatography and ultra-high resolution FT-MS proteomic analysis to analyze the *ex- vivo* expression of collagen fibril organizing proteins in normal esophageal fibroblasts (NOFs) compared with CAFs isolated from EAC that strongly expressed stromal SMA (Figure 7A). CAFs were found to maintain a myofibroblast phenotype in culture: shown by significantly increased SMA protein expression (*p* = 0.03; Figure S6A); and the formation of SMA positive stress fibers in > 70% of cells (Figure S5B). Unsupervised hierarchical clustering showed that the proteins formed two discrete clusters: cluster 1 (comprising of GREM1, COL14A1, CYP1B1, DDR2, LUM, TGFBR2, MIA3 & LOXL2) and cluster 2 (comprising of COL1A1, COL1A2, COL3A1, P4HA1, LOX, LEPRE1, ANXA2, TNXB, PLOD3 & SERPINH1). NOFs formed a tight cluster with relatively uniform expression, predominantly up-regulating the proteins in cluster 1 with downregulation of cluster 2. In contrast the CAFs' expression profile was significantly heterogenic, particularly for expression of proteins in cluster 2, which includes the genes up-regulated in EAC compared to normal tissues from the TCGA data (Figure 1: *COL1A1, LEPRE1, PLOD3, SERPINH1 & ANXA2*). For example, CAF1807 had particularly high expression of each of these proteins; whereas CAF 506 clustered separately from the other CAF samples and down-regulated the expression of each of these proteins (Figure 7B). Similar heterogeneity was found in mRNA expression in public datasets analyzing the gene expression profiles of CAFs from HNSCC and CRC (Figure S5C).

To determine whether the heterogeneous expression of these proteins *in vitro* correlated with the variation in collagen fiber elongation observed in SMA positive tumors *in vivo*, we performed SHG imaging of the tumors from which CAF1807 and 506 had been extracted. This showed that the CAF1807 tumors had significantly (*p* = 0.0016) elongated collagen fibers compared to the CAF 506 tumors, corresponding with the elevated expression of CFOGs from cluster 2 (Figure 7C–7D).

### Combined analysis of CAF's and collagen morphology provides refined prognostication

To determine whether the sub-set of SMA-positive CAFs with the ability to modify collagen structure impacted on patient prognosis we repeated the survival analysis by sub-dividing patients with an SMA positive tumor stroma according to the presence or absence of elongated collagen fibers. In each tumor type this significantly refined patient stratification (Figure 7E–7G; Log Rank *p* < 0.003).

## DISCUSSION

Our data illustrates the importance of myofibroblasts/CAF's role in manipulating the ECM architecture within the tumor microenvironment. We show that the formation of elongated collagen fibers within the tumor stroma predicts for poor patient survival rates in HNSCC, EAC and CRC. Furthermore, our data show that the formation of this collagen structure is regulated by a sub-set of SMA- positive CAFs, highlighting that the SMA-positive CAF phenotype is functionally heterogeneous.

There is limited research into the role and effect of CAFs on collagen re-modeling within the tumor microenvironment. *In vitro* studies have shown that stromal cells isolated from both breast and pancreatic tumors can create increasingly aligned fibronectin matrices in a syndecan-1 or FAP-dependent manner respectively, which facilitate tumor cell motility [23, 24]. Additionally, a breast cancer model system has shown that fibroblasts over-expressing LOX increase tumor cell invasiveness *in vivo*, by increasing matrix stiffening and focal adhesion formation [2]. It is likely that the cross-linking effects of LOX enzymes are involved in the formation of elongated collagen fibers observed in this study, as LOXL2 was one of the genes identified to be up-regulated in HNSCC, CRC and EAC (Figure 1D). Indeed, similar structural changes were observed in the study analyzing the effect of LOX enzyme expression in fibroblasts, on collagen structure [2]. Furthermore, our data has shown that LOXL2 was up-regulated in the TGF-β1 treated fibroblasts that regulate elongated collagen fiber formation *in vivo* (Figure 6E). A recent study using an orthotopic non-small cell lung cancer model has shown that stromal expression of integrin ^a11β1^ is critical for CAF differentiation and collagen reorganization *in vivo*, and this significantly affects metastatic potential [25].

The altered collagen morphology identified in this study is likely to be important in controlling tumor cell invasion and motility. Collagen structure has been shown to be prognostic in breast cancer [6], and a number of studies have shown that aligned matrices can affect the track-dependent migration of cancer cells [8, 9, 24, 26]. Increased expression of type I collagen and related genes is also frequently observed in the gene expression signatures associated with increased risk of metastasis [27, 28].

Cancer cell invasion through a 3D matrix is complex: requiring the generation of a tractive force between the cell and ECM [21], which is affected by matrix stiffness and proteolytic activity [29]. Notably, linearized fibers are stiffer than those with a less structured morphology and the resulting increased ECM stiffness can significantly promote cell migration [2, 29, 30], activating integrin-dependent mechanotransduction signaling pathways [31]. Furthermore, collagen remodeling has been shown to be associated with local cancer cell invasion, and intravital multi-photon microscopy has demonstrated that tumor cells migrate rapidly along collagen fibers in collagen-rich regions [9, 32–34].

It has also been suggested that collagen can act as a physical barrier to the invasion of cells through tissues [35], and the activity of proteolytic enzymes such as matrix metalloproteinases (MMPs) is critical for tumor invasion to occur [36–38]. In comparison the amoeboid nature of T-cell movement and their limited expression of MMPs is thought to prevent these cells from migrating through dense ECM [39, 40]. Therefore, alterations to the ECM may have a variable effect on different cell populations' motility within the tumor microenvironment, dependent on their method of movement, resulting in enhanced cancer cell invasion but limiting immune surveillance by preventing T-cell trafficking to the tumor [41].

In summary, recent studies have highlighted heterogeneity within the CAF population [42–46]. Our study reveals significant heterogeneity even within the SMA-positive ‘myofibroblastic’ CAF population, particularly related to collagen remodeling. The prognostic significance across tumor types of an SMA-positive stroma with elongated collagen fibers reveals a sub-set of CAFs associated with aggressive disease, and suggests that combined evaluation of collagen structure and stromal SMA expression may be highly relevant for the pathological and prognostic assessment of cancer [47, 48].

## MATERIALS AND METHODS

### Patient tissues

Tissue microarrays (TMAs) were constructed from previously described cohorts of HNSCC, EAC and CRC patients [18–20] from archival paraffin-embedded material at University Hospital Southampton (UHS), using randomly selected, 1 mm cores (Alphelys MiniCore 3). All tissue collection and storage was handled by a HTA (Human tissue authority) licensed tissue bank, with ethical approval and informed consent obtained (Rec No. 10/H0504/32 & 09/H0504/66). Baseline clinicopathological features and treatment details are shown in Supplementary Table 1.

### SHG Imaging and prognostic correlation testing

Fibrillar collagen was imaged using a custom-built multi-photon laser scanning microscope at the Laboratory for Optical and Computational Instrumentation (LOCI) University of Wisconsin-Madison. A Ti-sapphire (Spectra Physics Mai Tai) laser tuned to 890 nm was focused onto the sample using a 20x air immersion objective (Nikon S Fluor, N.A. = 0.75). Backscattered SHG signal was isolated using a 445–20 band-pass filter (Semrock). All images were acquired using WiscScan, an image acquisition software package developed by LOCI (http://loci.wisc.edu/software/wiscscan).

Collagen fiber analysis from SHG images was performed using CurveAlign (http://loci.wisc.edu/software/curvealign) and CtFIRE (http://loci.wisc.edu/software/ctfire), software packages designed at LOCI, as described previously [16]. 512 × 512 pixel images (457 μm^2^ area) were analyzed using each program. For collagen fiber angle measurements CurveAlign analysis was performed using default parameters. For measurements of collagen fiber length CtFIRE analysis was carried out using slight modifications to the default parameters to allow optimal image segmentation: a pixel intensity threshold of 25 was implemented to omit background noise; and the percentile of remaining curvelet coefficients was set to 0.3.

For human tissue analysis Hematoxylin & Eosin (H & E) or SMA IHC stained TMAs were used to collect SHG images (20X magnification). For the TMAs constructed from HNSCC and EAC cohorts a representative 457 μm^2^ SHG field of view was collected from stromal regions within each tumor core (3–6 tumor cores per patient) and analyzed. For the CRC TMA, large SHG fields of view were collected on the multiphoton using tiling acquisition features built into the WiscScan acquisition system and then loaded into FIJI for stitching [22, 49]. Imaging was carried out blinded to clinical outcome.

The maximal Youden's index (Sensitivity + Specificity −1) for classifying patients with short survival rates (cancer specific survival < 3 years) was used as a cut-off value for categorical survival analysis.

### Cell culture

Human fetal foreskin fibroblasts (HFFF2s) were obtained from the European Collection of Cell Cultures (ECACC; http://www.phe-culturecollections.org.uk) and cultured in Dulbecco's modified eagle medium (DMEM) supplemented with 2 mM L-Glutamine and 10% (v/v) fetal bovine serum (FBS) at 5% CO_2._

Adult primary fibroblasts were isolated from normal and EAC tissues (as described previously [18]) and cultured in Dulbecco's modified eagle medium (DMEM) supplemented with 2 mM L-Glutamine and 10% (v/v) fetal bovine serum (FBS) at 10% CO_2_. The fibroblastic phenotype was characterized by Western blotting to confirm vimentin expression and the absence of pan-cytokeratin/CD31 expression. The source of each NOF and CAF is denoted by a numeric label identifying the patient these cells were extracted from and clinicopathological details of the tumors that the CAFs were extracted from can be found in Supplementary Table 3.

5PT squamous cancer cells (a cisplatin resistant clone derived from the UM-SCC-5 parental cell line (20), were kindly provided by Prof Ian C. Mackenzie (Barts and the London School of Medicine and Dentistry, UK)).

Cells were cultured in Dulbecco's modified eagle medium (DMEM) supplemented with 2 mM L-Glutamine and 10% (*v/v*) fetal bovine serum (FBS). Treatment with 2 ng/ml of human recombinant TGF-β (R & D Systems) for 72 hours was used to induce HFFF2 myofibroblast differentiation. All cell lines were routinely tested for mycoplasma contamination.

### Fluorescence microscopy

1 × 10^4^ fibroblasts were plated on permanox chamber slides (8 chambers/slide; Thermo Scientific) in serum-free DMEM. Cells were fixed (4% w/v PFA), permeabilised (PBS + Triton-X100 (v/v 0.5%) and immunofluorescence for SMA (Sigma) performed using an Alexa 564-conjugated anti-mouse secondary antibody (Invitrogen) Nuclei and actin filaments were visualized using DAPI (1 μg/ml; Molecular Probes) and Phalloidin-FITC (0.5 μg/ml; SIGMA) respectively. Images were taken using an Olympus IX81 fluorescence microscope with a 20X air immersion objective (Olympus UPFLN, N.A. = 0.5).

### Gel contraction

2 × 10^5^ HFFF2 fibroblasts were mixed with 10X DMEM (+ 440 mM sodium bicarbonate); sterile H_2_O; and collagen, type 1, rat tail (Millipore) to form a 1 mg/ml collagen gel (1 ml total volume) [18]. Gels were imaged after 24 hours. The degree of gel contraction was quantified by measuring gel area (using FIJI [22]).

### qPCR

RNA was extracted using the RNeasy kit (Qiagen) and reverse transcribed using the RevertAid First Strand cDNA Synthesis Kit with oligodT primers, as per manufacturer's instructions. PCR was performed using 10 ng of cDNA, 2X SYBR green mastermix (Applied Biosytems) and primers (described below) under the following conditions: 95°C hold step for 10 minutes, followed by 40 cycles of 95–60°C for 15 seconds and 5 minutes respectively. Relative levels of mRNA expression were determined using the ddCt method. Primer sequences used were as follows: *ACTB* (forward TGGCACCCAGCACAATGAA, reverse CTAAGTCATAGTCCGCCTAGAAGCA) as a housekeeping gene; *COL1A1* (forward ACGAAGAC ATCCCACCAATCACCT, reverse AGARCACGTCATC GCACAACACCT); *COL3A1* (forward AATCAGGTAGA CCCGGACGA, reverse TTCGTCCATCGAAGCC TCTG); GAPDH (forward AGCAATGCCTCCTGCACCA CCAAC, reverse CCGGAGGGGCCATCCACAGTCT); VIM (human not mouse; forward GGACCAGCTAA CCAACGACA, reverse GCAGCTCCTGGATTTCCTCT).

### Xenograft model

All experiments were reviewed and approved by both the Science Review Group and the Animal Welfare and Ethical Review Board, University of Southampton, and were carried out under UK Home Office license number PPL30/3028. Animals were obtained from Charles River Laboratories; they were bred and housed in a local animal facility and used between 8–12 weeks of age.

1 × 10^6^ 5PT ± 3 × 10^6^ HFFF2s were suspended in 150 ml of DMEM + 2 mM L-Glutamine. 100 ml of this mix was injected subcutaneously (s.c.) into the flank of partially immunocompromised C57BL/6 RAG1^−/−^ male mice. Animals were euthanized after 5 weeks. Tumors were removed and fixed in 10% formalin, processed to paraffin and H & E stained. Three independent stromal regions per tumor were imaged by SHG and analyzed using CurveAlign and ctFIRE as described above.

### Immunohistochemistry

Automated immunostaining of TMAs was performed in the UHS Clinical Cellular Pathology Laboratory (anti-SMA antibody; M0851, Dako). Staining was evaluated using a semi-quantitative scoring system, as described previously [10], according to the extent of stromal positivity (low/negative [< 5% stroma positive], moderate [patchy/focal expression, 5–50% stroma positive] or high [diffuse expression throughout tumor, > 50% stroma positive]. Scoring was carried out independently by (GJT & TJU) blinded to clinical outcome.

### Gene expression profiling analysis

Gene expression profiling data was from the following publicly available databases was used in this study. Figure 1: RSEM normalized results from RNASeq data for Patient Matched normal and tumor tissues (HNSCC, EAC and CRC TCGA). Figure S5C: RMA normalized data from affymetrix array profiling of CAFs isolated from HNSCC (GSE38517) and CRC (GSE46824). The data was Log transformed (unless this had already been carried out prior to upload in the databank) and genes within the collagen fibril organization gene ontology term (GO:0030199) were extracted, using the maximal value where multiple probes identified the same gene. Gene pattern software was used for downstream analyses [50]. Hierarchical clustering was carried out with sample distance measured using a Euclidean distance measure and gene distance by Pearson's correlation [51]. Differential expression was assessed between normal and tumor samples using comparative marker selection [52].

### Proteomics

Specimens were homogenized in 200 μL of dissolution buffer (0.5 M TEAB, 0.05% SDS) using the FastPrep system (Savant Bio, France) and pulsed probe sonication. Lysates were centrifuged (16, 000 g, 10 min, 40C) and supernatants measured for protein content (BCA assay; Thermo Pierce, Rockford, IL, USA). A total of 100 mg final protein content from each specimen was subjected to reduction, alkylation, trypsin proteolysis and 8-plex iTRAQ labeling with the following reporter ion / sample ID assignment: 113/NOF-EN102; 114/NOF2009; 115/NOF-EN251; 116/CAF1807; 117/NOF1807; 118/CAF0801; 119/CAF255; 121/CAF506. The labelled peptide mixtures were pooled and offline separated with high-pH reverse phase (RP) linear gradient chromatography using the Waters, XBridge C8 column (150 × 3.0 mm, 3.5 mm particle) with the UltiMate nano-HPLC system generating a total of 30 fractions (Dionex, Sunnyvale, CA, USA) [53]. Each fraction was analyzed using LC-MS with low-pH RP capillary chromatography (PepMap C18, 50 μm ID × 50 cm L, 100 Å pore, 3.5 μm particle) and nanospray ionization FT-MS (Dionex Ultimate 3000 UHPLC system - LTQ-Velos Pro Orbitrap Elite, Thermo Scientific, USA) [53, 54].

Unprocessed raw files were submitted to Proteome Discoverer 1.4 for target decoy searching with SequestHT for tryptic peptides, allowing two missed cleavages, a tolerance of 10 ppm, a minimum peptide length of 5, and a maximum of 2 variable (1 equal) modifications for: oxidation (M), deamidation (N, Q), or phosphorylation (S, T). Fixed modifications included: Methythio (C) and iTRAQ (K, Y, and N-terminus). Fragment ion mass tolerances of 0.02 Da for the FT-acquired HCD spectra and 0.5 Da for the IT-acquired CID spectra. FDR was estimated with the Percolator and set to ≤ 0.01 and validation was based on *q* value at < 0.01. Reporter ions were extracted with a tolerance of 20 ppm and were omitted if any channels were absent. Quantification ratios were median-normalized and transformed to the log2 scale. Spectra were searched against the UniProtKB TREMBL mouse proteome (Dec 2013).

### Statistical analysis

Statistical analysis of patient survival rates was carried out using SPSS v22 (IBM SPSS Inc., Chicago, IL, USA). The primary endpoint was death from cancer, survival time was measured from the date of diagnosis in the HNSCC cohort and date of surgery in the EAC and CRC cohorts. Other causes of death were censored at the time of death. Kaplan-Meier plots (with Log-rank [Mantel-Cox] tests) and unadjusted Cox proportional hazards models were used to describe the risk of dying from cancer within the indicated stratification metrics, unless otherwise stated.

Statistical testing between groups and treatment conditions was carried out using unpaired 2-tailed homoscedastic *T*-tests, as described in relevant figure legends. *p < *0.05*, **p < *0.01*, ***p < *0.001* and ****p < *0.0001*.

## SUPPLEMENTARY MATERIALS FIGURES, TABLES


