# Supplementary material for: A subset of myofibroblastic cancer-associated fibroblasts regulate collagen fiber elongation, which is prognostic in multiple cancers
Source: Oncotarget. 2015 Dec 23;7(5):6159–74. doi: 10.18632/oncotarget.6740 (PMC4868747; doi:10.18632/oncotarget.6740)
Supplement: Supplementary file 1 [file oncotarget-07-6159-s001.pdf]

A subset of myofibroblastic cancer-associated fibroblasts regulate collagen fiber elongation, which is prognostic in multiple cancers

Supplementary Materials

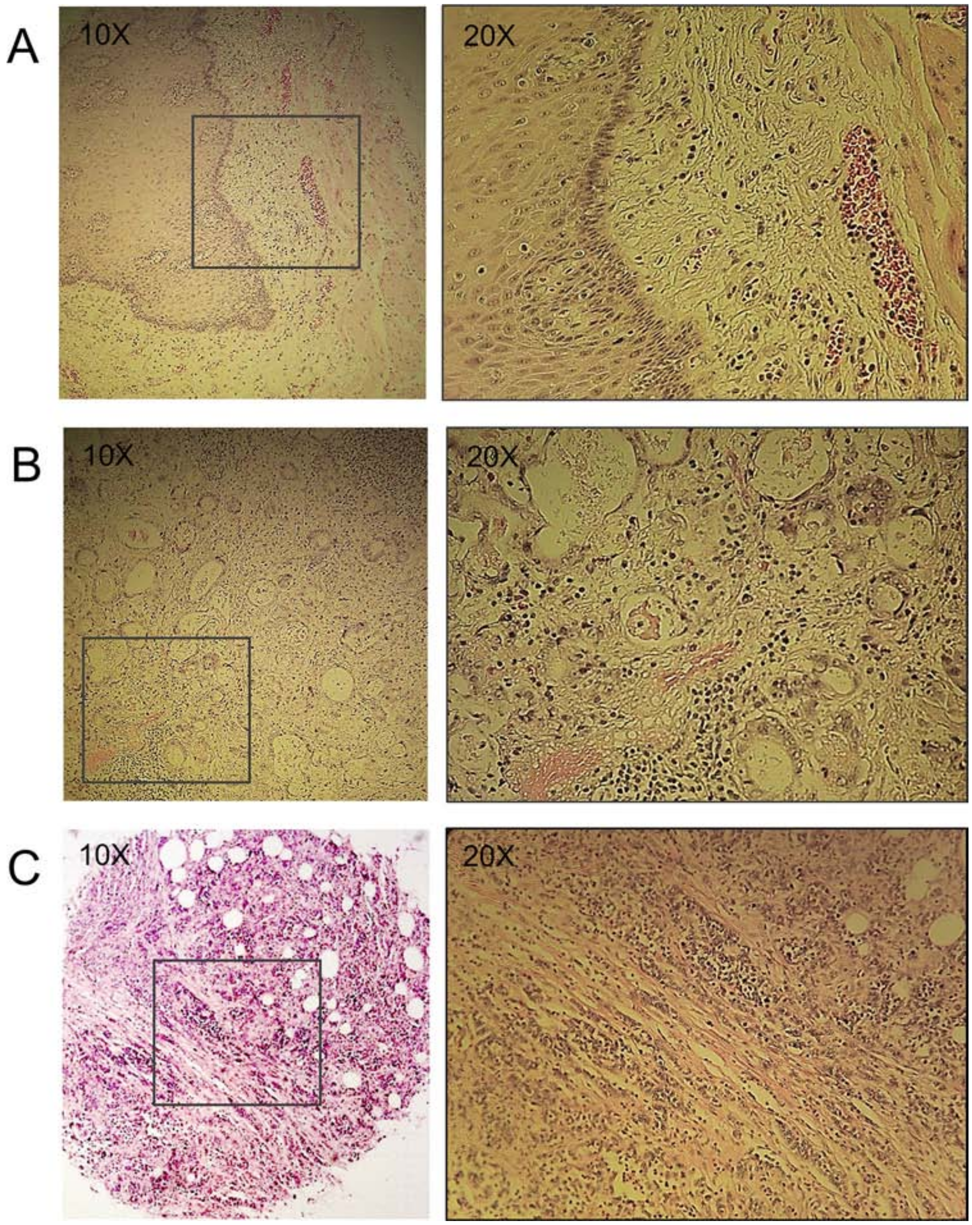

Supplementary Figure S1: Low magnification H&E stained images of sections presented in Figure 2 **(A–C).** Normal squamous epithelium and sub-epithelial stroma (A) Sub-mucosal tissue (B) from esophageal tissue samples. (C) Esophageal adenocarcinoma (EAC) tissue.

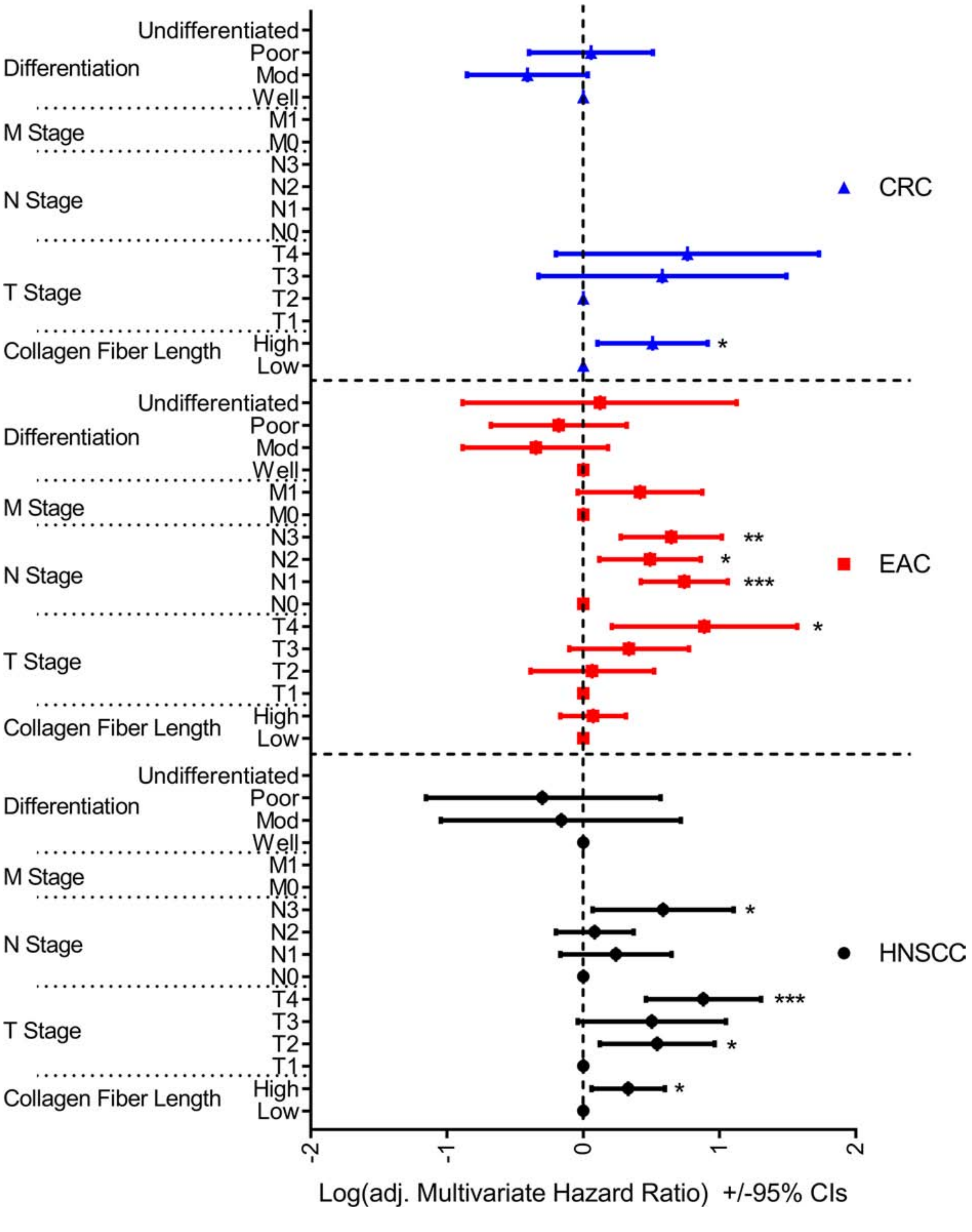

Supplementary Figure S2: Multivariate Cox Regression analysis of survival in the HNSCC, EAC and CRC cohorts adjusted for Collagen fiber length, T/N/M staging and differentiation.

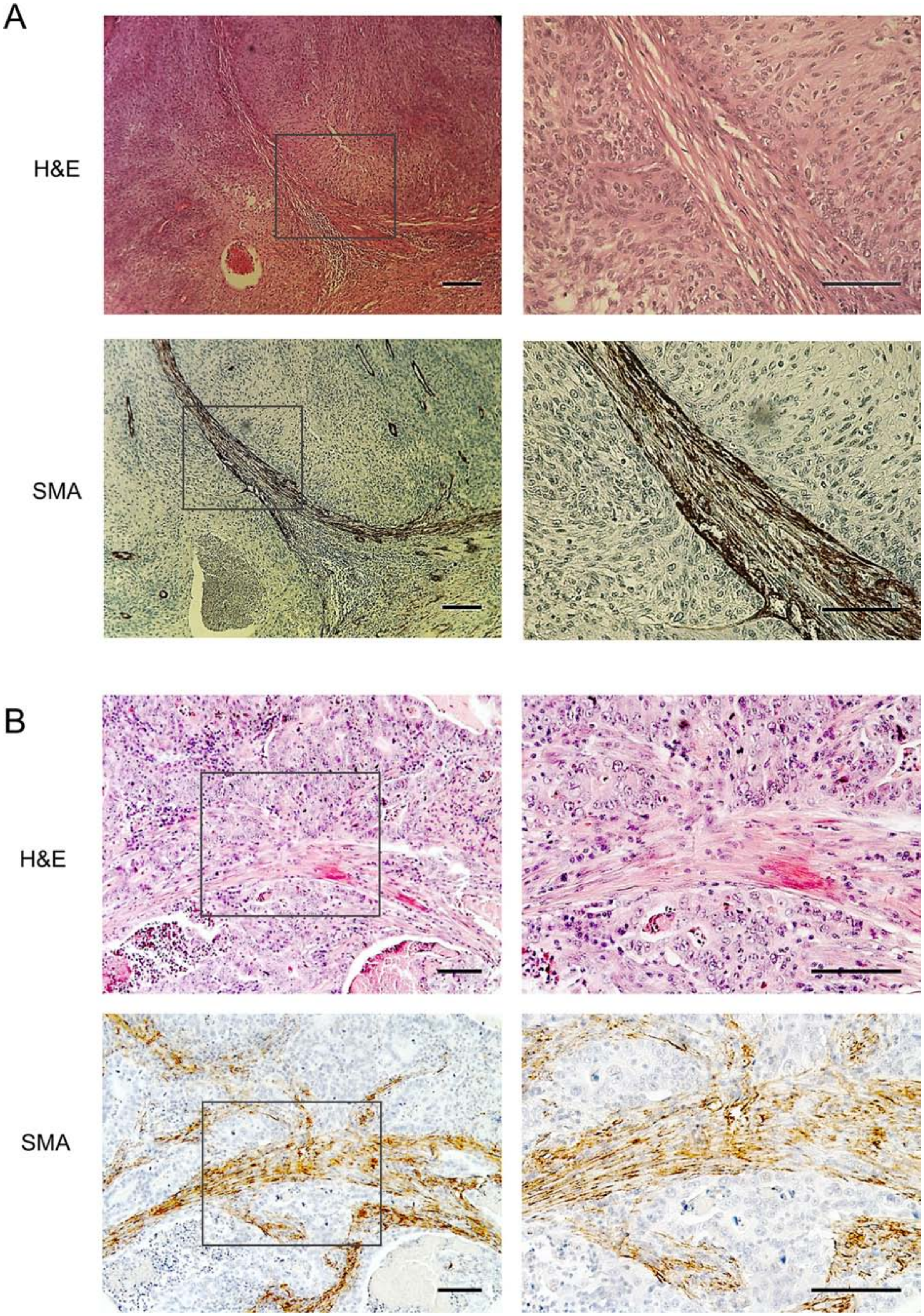

Supplementary Figure S3: Low magnification H & E and SMA stained images of sections presented in Figure 6A: (**A**) HNSCC tissue and (**B**) EAC tissue. Scale Bars represent 100 μm.

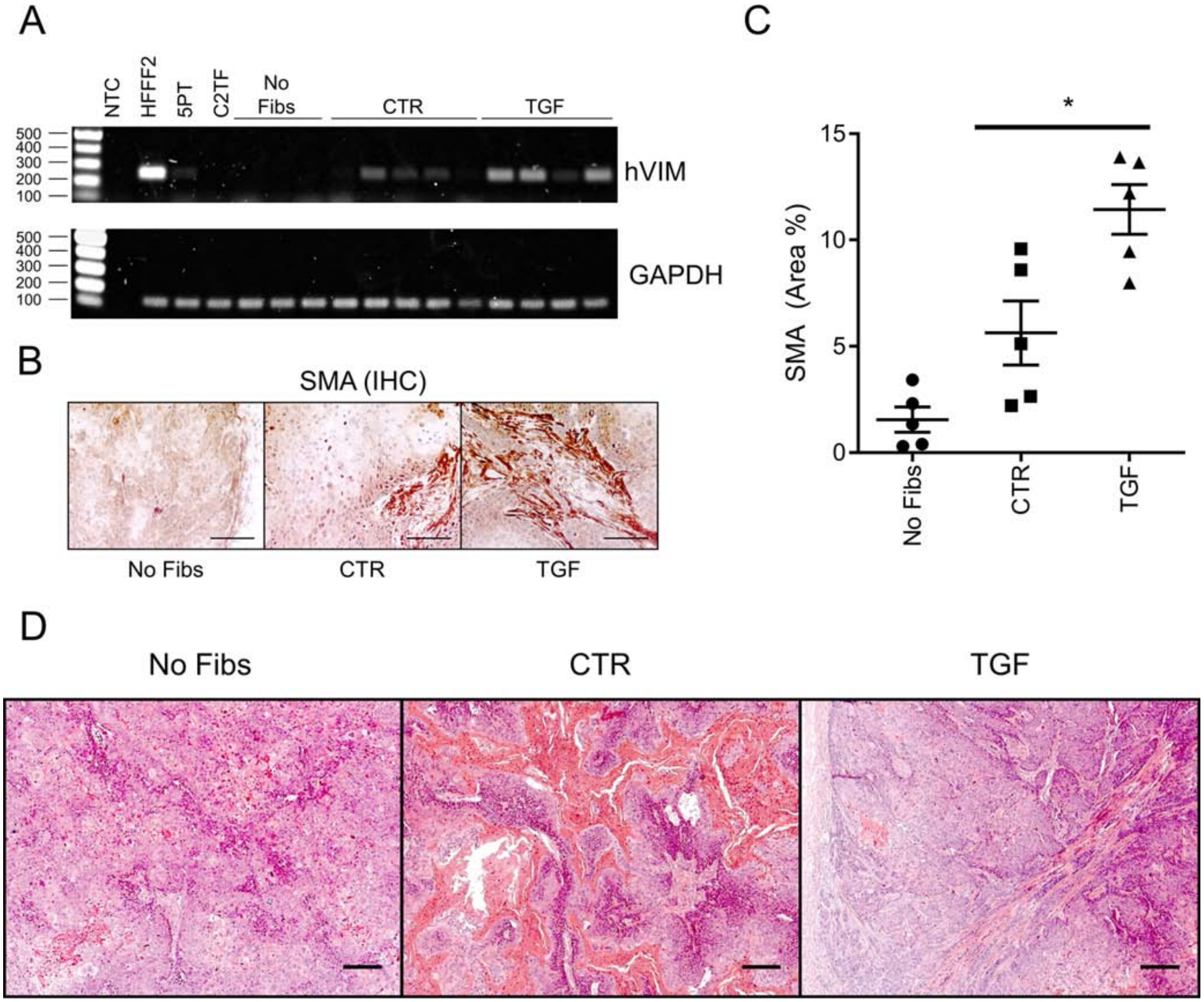

Supplementary Figure S4: Analysis of human fibroblast persistence in 5PT xenograft model. (**A**) Agarose gel electrophoresis showing end-point PCR (40 cycles) of human vimentin (hVIM) mRNA expression, GAPDH was used as a housekeeping gene. Sample descriptions: 5PT – Cancer cell line; C2TF – murine fibroblasts; No fibs – Xenografts injected with 5PT cells alone; CTR/TGF – Xenografts injected with 5PT cells plus control or TGF-β treated HFFF2s respectively. (**B**) SMA Immunohistochemistry staining of 5PT xenograft tumors. (**C**) Histogram showing SMA positive area in each tumor type. SMA positive area was quantified using the "Trainable Weka segmentation" plugin in Fiji. Data is presented as the mean positive area (%) per tumor from 5 20x fields of view. Statistically significant differences were assessed by 2-tailed, unpaired, homoscedastic *T*-test (*$p < 0.05$). (**D**) Low magnification H&E stained images of sections presented in Figure 6F. Scale bars represent 100 µm.

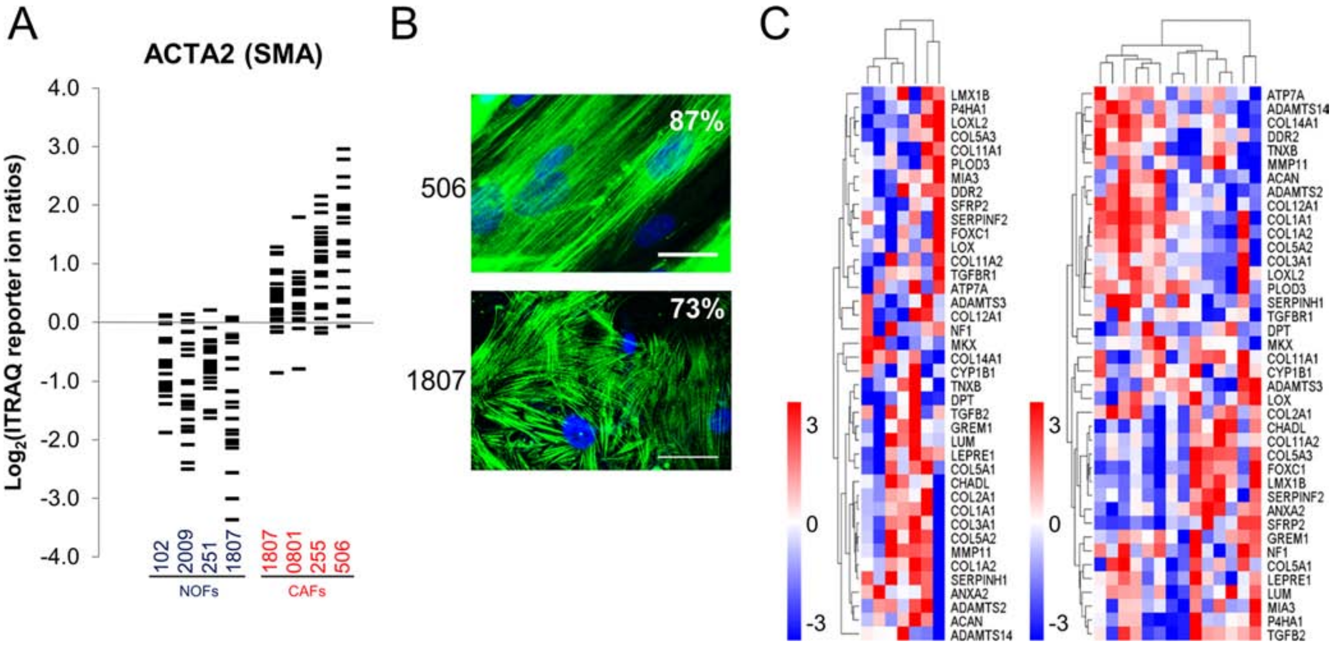

Supplementary Figure S5: Heterogenic expression of CFOGs by myofibroblastic CAFs cultured *ex-vivo*. (**A**) iTRAQ reporter ion ratios derived from 11 surrogate peptides observed in 23 peptide spectral matches (PSMs) for ACTA2 (SMA), demonstrating its overall higher relative expression in each CAF compared to NOFs (*T*-test $p = 0.03$). (**B**) Immunocytochemistry staining for SMA (Green) and DAPI (Blue) in CAF 506 and 1807, the percentage of SMA positive cells (from 6 fields of view) is shown in the top right corner of each image. Scale bar represents 50 µm. (**C**) Unsupervised hierarchical clustering of CAFs isolated from HNSCC (Left) or CRC (Right), using a Euclidean distance measure of pairwise average-linkage based on mRNA expression of CFOGs. Expression levels are row normalized for visualization.

Supplementary Table S1: Baseline clinical and histopatholological features of patients from each cohort.

		HNSCC	EAC	CRC
**Number**		213	146	64
**Median age at diagnosis (range)**		57 (28−90)	67 (36−85)	73 (39−89)
**Median length of follow-up (months)**		36	27	54
**Cancer related deaths**		59	67	25
**Gender**	Male	153	124	48
	Female	60	22	16
**T Stage**	Tis	1		
	T0	0	9	
	T1	51	31	
	T2	88	36	4
	T3	21	67	41
	T4	58	3	19
	Tx	3		
**N stage**	N0	49	74	64
	N1	26	32	
	N2	128	23	
	N3	8	17	
**M stage**	M0	211	141	64
	M1	1	5	
**TNM**	Missing	1	0	0
**Grade of differentiation**	Well Differentiated	2	13	23
	Moderately Differentiated	71	43	28
	Poorly Differentiated	139	89	13
	Undifferentiated		1	
	Missing	1	0	0
**Treatment of Primary Tumour**	Surgery only	78	60	46
	Adjuvant Chemo/Radio/Chemoradio Therapy	95		10
	Neoadjuvant Chemo/Radio/Chemoradio Therapy	40	86	2
	Adjuvant & Neoadjuvant Chemo/Radio/Chemoradio Therapy			6

Supplementary Table S2: Cross-tabs showing SMA status and corresponding collagen fiber length.

			Collagen Fiber Length	
			**Low**	**High**
**HNSCC**	**SMA**	Low	47	41
		Mod	21	62
		High	9	21
		Totals	77	124
**EAC**	**SMA**	Low	6	2
		Mod	34	39
		High	19	29
		Totals	59	70
**CRC**	**SMA**	Low	11	6
		Mod	15	10
		High	8	5
		Totals	34	21

Supplementary Table S3: Clinicopathological data on tumours from which the CAFs were extracted

	**116/CAF1807**	**118/CAF0801**	**119/CAF255**	**121/CAF506**
**Age**	58	61	64	65
**Sex**	Male	Male	Male	Male
**Neoadjuvant chemotherapy**	No	Yes	Yes	No
**Response to neoadjuvant**	N/A	No response	Yes – minimal residual tumour	N/A
**Tumour grade**	Moderately differentiated adenocarcinoma	Moderately differentiated intestinal-like adenocarcinoma	Poorly differentiated adenocarcinoma	Poorly differentiated adenocarcinoma
**Regional metastasis**	Yes	Yes	No	Yes
**Distant metastasis**	No	No	No	No
**Pathology stage**	PT3N3	PT3N1	PT3N0	PT3N2
